# Neuropsychiatric Effects in Patients With Invasive Prolactinomas Treated With Cabergoline

**DOI:** 10.7759/cureus.39869

**Published:** 2023-06-02

**Authors:** Metztli Calva-González, Pedro Leonardo Villanueva-Solórzano, Edgar D Crail-Meléndez, Kennya M Loya-Murguia, Itzel Ariadna Dehesa Hernandez, Fernando Robles-Ramirez, Luis A Rodríguez-Hernández, Michel G Mondragón-Soto, José Guillermo Flores-Vázquez, Lesly A Portocarrero-Ortiz

**Affiliations:** 1 Psychiatry, National Institute of Neurology and Neurosurgery Manuel Velasco Suárez, Mexico City, MEX; 2 Neurosurgery, National Institute of Neurology and Neurosurgery Manuel Velasco Suárez, Mexico City, MEX; 3 Neuropsychiatry, National Institute of Neurology and Neurosurgery Manuel Velasco Suárez, Mexico City, MEX; 4 Neuroimaging, National Institute of Neurology and Neurosurgery Manuel Velasco Suárez, Mexico City, MEX; 5 Neuroradiology, National Institute of Neurology and Neurosurgery Manuel Velasco Suárez, Mexico City, MEX; 6 General Surgery, Centro Medico ABC, Mexico City, MEX; 7 Radioneurosurgery, National Institute of Neurology and Neurosurgery Manuel Velasco Suárez, Mexico City, MEX; 8 Neuroendocrinology, National Institute of Neurology and Neurosurgery Manuel Velasco Suárez, Mexico City, MEX

**Keywords:** pituitary adenoma. brain tumor, cabergoline, adverse effects, neuropsychiatric, prolactinoma

## Abstract

Background and objective

Invasive prolactinoma accounts for 1-5% of all prolactinomas. Its mass and compromise of the diencephalon and frontal and temporal lobes may result in a range of neuropsychiatric symptoms that are often missed during initial evaluations. Cabergoline is a dopaminergic agonist used as the first-line treatment for these patients; however, its effect on neuropsychiatric symptoms in this particular setting remains unexplored. In this study, our primary objective was to describe the epidemiology of neuropsychiatric comorbidities in Mexican patients with invasive prolactinomas. The secondary aim of the study was to describe how these comorbidities are modified by treatment with cabergoline, through follow-up with standardized clinical scales.

Methods

This was a retrospective analytic study. Data were pulled from clinical records and evaluations of patients at baseline and at six-month follow-ups.

Results

A total of 10 patients were included in the study. None of them had any prior psychiatric diagnosis. At the initial evaluation, 70% were diagnosed with depression or anxiety. During follow-up, two patients developed neuropsychiatric symptoms; there was a significant reduction in tumor size but no difference was found in clinimetric scores for neuropsychiatric comorbidities.

Conclusions

Patients with giant prolactinomas may present with several neuropsychiatric symptoms throughout the course of their disease. Although there are several mechanisms involved, it is important to keep in mind that cabergoline may interfere with the dopaminergic pathways involved. This study was underpowered to determine the association but can serve as a pilot for further research on this topic.

## Introduction

Pituitary adenomas are common tumors, accounting for 10-20% of intracranial neoplasms and ranking as the third most frequent neoplasm after gliomas. Their estimated prevalence is around 80-90 cases per 100,000 individuals [1,2]. Approximately 40% of pituitary adenomas originate from lactotroph cells and are known as prolactinomas [2,3,4]. Prolactinomas can be classified based on their size into microprolactinomas (diameter <10 mm), macroprolactinomas (diameter >10 mm), and giant prolactinomas (diameter >40 mm). Giant prolactinomas constitute 1-5% of all prolactinomas. In terms of gender distribution, while micro and macroprolactinomas are more common in women, giant prolactinomas are more prevalent in men, with a male-to-female ratio of 9:1 [2]. Due to their larger size, giant prolactinomas can exhibit invasive behavior and cause symptoms such as frontal disinhibition, disorientation, reversible cognitive impairment, hemiparesis, olfactory hallucinations, temporal lobe epilepsy, cerebrospinal fluid (CSF) nasal fistula, epistaxis, exophthalmos, cranial nerve involvement (III, IV, V, VI), and hydrocephalus [2,3,4].

The treatment primarily aims to normalize or reduce prolactin hormone levels, decrease tumor size, improve focal neurologic symptoms caused by compression, achieve eugonadism by normalizing testosterone levels, and restore the hormonal axis affected by the tumor. The Society of Endocrinology recommends cabergoline as the first-line medical treatment for these tumors. Cabergoline is classified as an ergotamine-derived dopaminergic agonist. Compared to other drugs in this category, cabergoline has demonstrated greater therapeutic effectiveness due to its high selectivity for D2 receptors and its long half-life of approximately 65 hours. It also has a lower incidence of adverse effects [3]. The most common adverse effects include nausea, orthostatic hypotension, headache, Raynaud's phenomenon, nasal congestion, and constipation. Less frequently, valvular heart disease and neuropsychiatric symptoms such as impulse control disorders, depression, anxiety, psychosis, and mania have been reported, either as new effects or exacerbations of pre-existing symptoms [5]. There is a lack of evidence regarding the incidence and prevalence of these manifestations in patients with prolactinomas, and no studies have been conducted in the Mexican population.

We believe the findings of the present investigation will provide valuable insights into the incidence of neuropsychiatric manifestations in a specific population. This information can guide the proactive screening for these clinical symptoms in prolactinoma patients receiving cabergoline, with the aim of improving clinical care efficiency and safety for these patients.

## Materials and methods

A prospective observational analytic study was conducted between January 2020 and July 2021, with a minimum follow-up of six months. A sample size of 15 was initially calculated according to local epidemiology. Patients aged 18-70 years, with confirmed invasive prolactinoma (through clinical investigations, neuroimaging, and hormone profile) treated at the National Institute of Neurology and Neurosurgery Manuel Velasco Suárez were included. Patients with nonfunctioning macroadenomas, acromegalia, Cushing disease, TSH-producing macroadenomas, or other middle-line lesions were excluded. Written informed consent was obtained from all participants who agreed to participate, and patients who declined consent were excluded.

A total of 16 patients met the inclusion criteria; however, four of them declined consent, and two of them were later found to have a different diagnosis (one of them had acromegalia and the other one had mixed histology). Ultimately, 10 patients were included for analytic purposes. A multidisciplinary review of all diagnostic tests (1.5 T MRI, serum prolactin levels, extended hormone profile) with the participation of a neurosurgeon, a neuroendocrinologist, and a neuroradiologist was performed to confirm the diagnosis. To decide whether the lesion was invasive or not, we used the Hardy criteria for extrasellar extension and/or Knosp grade 2 or above.

All patients received baseline psychiatric evaluation performed by a psychiatrist, which consisted of a neuropsychiatric interview, complete neurologic exam, and application of the following clinimetric scores: Mini International Neuropsychiatric Interview (MINI); Hamilton Depression Rating Scale (HAM-D); Beck Depression Inventory (BDI); Hamilton Anxiety Rating Scale (HAM-A); Changes in Sexual Functioning Questionnaire (CSFQ); Barrat Impulsiveness Scale (BIS).

BDI was used for self-reported outcomes, through a questionnaire in the waiting room, and the Hamilton Depression Rating Scale for clinician-reported outcomes. For comparison purposes, the HAM-D scale was used. Patients were followed up with clinical scores and serum prolactin levels at one, three, and six months. A repeat MRI was performed at the six-month follow-up.

A univariate analysis was performed using parametric [mean, standard deviation (SD)] and non-parametric analyses. Baseline characteristics and outcome variables were compared between both groups using a student's t-test for parametric continuous variables and a Chi-squared test for categorical variables. A Pearson correlation test was performed to assess the association between continuous variables.

All procedures described above were conducted in accordance with the regulations established in the "Regulation of the General Health Law regarding Health Research" under "Title Two, Chapter I, Article 17: Section I - Non-Risk Research." Additionally, the study adhered to ethical guidelines, including the World Medical Association's Declaration of Helsinki, the Council for International Organizations of Medical Sciences (CIOMS), and the International Ethics Guidelines. This article received approval from the Research Ethics Committee of the Manuel Velasco Suarez National Institute of Neurology and Neurosurgery with approval number 74/20.

## Results

Case summaries

Case 1

A 46-year-old male with prior retinopathy presented to the emergency department in February 2021 with symptoms of intracranial hypertension, and obstructive hydrocephalus secondary to a midline lesion was documented. A ventriculoperitoneal shunt was placed. Initial PRL was 9,559 ng/ml; cabergoline was started at 0.5 mg qd and adjusted up to 1.5 mg qd on follow-up. Upon baseline evaluation, he was also diagnosed with generalized anxiety disorder, and hence sertraline 50 mg was started. At the six-month follow-up, neuropsychiatric symptoms had resolved and adequate PRL suppression was achieved. Sertraline was then discontinued.

Case 2

A previously healthy 47-year-old female presented to the emergency department in December 2021 with unequivocal signs of pituitary apoplexy, which was confirmed by MRI. Initial PRL was 30,010 ng/ml. She was treated with surgical resection. Upon baseline evaluation, she reported depressive symptoms, which she attributed to visual loss. She was started on cabergoline 0.5 mg qd, which was adjusted up to 1 mg qd on follow-up. At the six-month follow-up, her depressive symptoms had resolved and adequate PRL suppression was achieved. 

Case 3

A 31-year-old male presented to the neurology consultation with recent-onset seizures. He described a focal onset with behavior arrest and manual automatisms, suggestive of temporal localization. Bitemporal hemianopsia was documented, and later on, a giant prolactinoma was diagnosed. His initial PRL levels were 1,490 ng/ml. Treatment with carbamazepine 400 TID and cabergoline 0.5 qd was started. Although he became seizure-free, at the one-month follow-up, manic symptoms such as hypersexuality, agitation, suicidal ideation, increased energy, and grandiose plans were identified. He was admitted for surgical treatment of the tumor, and clozapine was started for symptom management. His symptoms resolved with pharmacologic treatment. Even though he had been receiving a low dose of the dopaminergic agonist, it is important to consider that his seizure semiology was localized to the temporal lobe, which could also contribute to the development of neuropsychiatric symptoms. At this time, we can only describe the episode and hypothesize about the possible physiopathogenic mechanisms.

Case 4

A 34-year-old male with a history of gastric tube placement due to sodium hydroxide poisoning presented with mesial temporal focal epilepsy and a sellar lesion. There were no campimetric abnormalities. An invasive macroprolactinoma with an initial PRL level of 863 ng/ml, grade IV obesity (BMI of 51.5), and hypogonadotropic hypogonadism were diagnosed. He developed a spontaneous CSF fistula that required surgical management. He continued to receive treatment and achieved appropriate control under the care of neuroendocrinology during follow-up.

Case 5

A 35-year-old male with no significant medical history presented with decreased visual acuity (bitemporal hemianopsia). An ophthalmological evaluation revealed an invasive macroprolactinoma, for which treatment was started in 2020 with an initial PRL level of 5,020 ng/ml. He is currently undergoing follow-up with neuroendocrinology and showing a satisfactory response to treatment.

Case 6

A previously healthy 25-year-old male presented to the ED with pituitary apoplexy secondary to a giant prolactinoma. His initial PRL level was 1,199 ng/ml; his thyroid axis was also compromised, and hence he was started on cabergoline at 0.5 mg qd and levothyroxine 75 mcg qd. Upon follow-up, poor compliance with treatment was identified. He reported depressive symptoms, decreased libido, and erectile dysfunction. He was referred to undergo cognitive behavioral therapy, which led to significant improvement in his adherence to treatment and hormone levels.

Case 7

A 40-year-old male with no significant medical history presented with sudden-onset decreased visual acuity, temporal headache, and an initial PRL level of 5,333 ng/ml. He was diagnosed with an invasive prolactinoma in 2020 and is currently followed up at a neuroendocrinology clinic, receiving appropriate hormone replacement therapy.

Case 8

A 35-year-old female with a history of drainage of renal abscesses in 2007 and 2015 (secondary to recurrent urolithiasis) presented with focal-onset epilepsy suggestive of right temporal lobe involvement and the finding of a right temporal meningoencephalocele on imaging. She was diagnosed with prolactinoma and treatment was started in April 2021 with an initial PRL level of 3,259 ng/ml. She is currently being followed up by endocrinology, showing a satisfactory response to dopamine agonist therapy with normalization of PRL levels. She is asymptomatic and also being followed up by the epilepsy clinic.

Case 9

A previously healthy 42-year-old male presented to the ED with signs of pituitary apoplexy, including vision loss. Initial PRL levels were 7,380 ng/ml. He underwent surgical resection with ventriculoperitoneal shunt placement. He was discharged on cabergoline 0.5 gm qd. At the one-month follow-up, he reported mild anxiety and insomnia. At six months, all mood changes had been resolved and cabergoline was discontinued because of adequate PRL suppression and lack of recurrence on MRI.

Case 10

A 50-year-old male with no significant medical history presented with superior temporal quadrantanopia in the right eye and temporal hemianopsia in the left eye. He was diagnosed with macroprolactinoma in November 2020, with an initial PRL level of 2,530. He also had central hypothyroidism and received replacement therapy. He was started on cabergoline 0.5 mg, resulting in visual improvement and symptom relief. He is currently being followed up by neuroendocrinology, with a reduced cabergoline dose of 50%, achieving appropriate tumor and hormonal control.

In summary, 10 patients were included for analytic purposes, and eight of them were male. All subjects were found to have neurological comorbidities such as visual loss, seizures, or cephalea. Even though none of our patients had reported previous psychiatric diagnoses, seven of them met the criteria for an affective disorder during baseline evaluation. Additional demographic characteristics are presented in Table 1.

**Table 1:** Patients’ characteristics before treatment (N=10) *Normal range adjusted to lab values of our institution (INNN): prolactin (ng/mL) [range: 3.7-17.9], TSH (IU/mL) [range: 0.46-4.68], cortisol (mcg/dL) [range: 4.46-22.7], LH (mUI/mL) [range: 2.8-6.8] *Number of affected areas evaluated by MRI head, detailed in Table 2 SD: standard deviation; TSH: thyroid-stimulating hormone; FSH: follicle-stimulating hormone; LH: luteinizing hormone; HDS: Hamilton Depression Scale

Characteristics	Values
Age in years, mean ± SD	34.60 ± 7.47
Male gender, n/N (%)	8/10 (80%)
Marital status, n/N (%)	
Married	3/10 (30%)
Single	7/10 (70%)
Smoking, n/N (%)	1/10 (10%)
Alcoholism, n/N (%)	0 (0%)
Cannabis, n/N (%)	1/10 (10%)
Previously diagnosed neurologic comorbidity, n/N (%)	10/10 (100%)
Previously diagnosed psychiatric comorbidity, n/N (%)	0 (0%)
Hormonal levels*, mean ± SD	
Prolactin, ng/mL	7871.90 ± 8946.48
TSH, mIU/mL	2.41 ± 1.28
Cortisol, mg/dL	17.22 ± 16.97
Testosterone, nmol/L	4.75 ± 5.45
FSH, mUl/mL	5.90 ± 2.38
LH, mUl/mL	3.84 ± 1.87
HDS severity classification, n/N (%)	
Without depression (0-6 points)	3/10 (30%)
Mild depression (7-17 points)	4/10 (40%)
Moderate depression (18-24 points)	3/10 (30%)
Severe depression (25-52 points)	0/10 (0%)
Prolactinomas dimensions, mean ± SD	
Pretreatment volume, cm^3^	36.20 ± 29.78
Rostrocaudal dimensions, cm	3.79 ± 1.28
Dorsoventral dimensions, cm	4.35 ± 1.47
Laterolateral dimensions, cm	3.99 ± 1.60
Prolactinoma classification, %	
Giant	90%
Mega giant	10%
Affected areas**, mean ± SD	16 ± 9.98

Regarding the hormonal axes, all patients exhibited significant hyperprolactinemia (7871.90 ± 8946.48), and testosterone levels were at the lower limit of normal, with a mean of 4.75 nmol/L [4.56-28.2]. The starting dose of cabergoline was 0.5 mg qd with adjustments made based on clinical response and follow-up PRL levels. The range dose for the study was 0.5-2 mg qd. At the beginning of the treatment, seven out of eight patients presented with hypogonadism, and at the end of the follow-up, four patients showed recovery while three continued to have the disorder. Additionally, three out of eight patients presented with hypothyroidism, and by the end of the follow-up, all of them had achieved euthyroid status.

MRI scans were performed to measure volume variables and quantify the areas of the brain affected by the tumor. As shown in Table 2, the frontal lobe and sellar region were the most affected cerebral areas due to the invasive behavior of these tumors. The scores of the initial and six-month follow-up tests are presented in Table 3. Prior to treatment, the initial HAM-D test indicated severe depression. The other scales did not provide a cutoff for severity classification.

**Table 2:** Most affected areas by the tumor, evaluated by MRI head MRI: magnetic resonance imaging

Affected areas	No. of patients with affected areas on MRI before treatment	No. of patients with affected areas on MRI six months after treatment
Frontal lobe		
Right frontal lobe compression	8/10	2/9
Left frontal lobe compression	9/10	1/9
Right rectus gyrus	8/10	2/9
Left rectus gyrus	9/10	1/9
Other structures		
Pituitary stalk (compression)	10/10	4/9
Optic chiasm (compression)	10/10	5/9
Sphenoidal sinus	10/10	1/9
Hypothalamus	10/10	4/9
Suprachiasmatic cistern (obliteration)	10/10	1/9
Third ventricle	9/10	2/9

**Table 3:** Pre-treatment and post-treatment hormonal levels *Normal ranges: prolactin (ng/mL) [range: 3.7-17.9]; TSH (IU/mL) [range: 0.46-4.68]; cortisol (mcg/dL) [range: 4.46-22.7]; testosterone (nmol/L) [range: 4.56-28.2]; FSH (mUl/mL) [range: 1.55-9.74]; LH (mUl/mL) [range: 2.8-6.8] SD: standard deviation; TSH: thyroid-stimulating hormone; FSH: follicle-stimulating hormone; LH: luteinizing hormone

Hormonal levels		
Hormone*	Pre-treatment, mean ± SD	Six months post-treatment, mean ± SD
Prolactin, ng/mL	7871.90 ± 8946.48	151.48 ± 148.71
TSH, mIU/mL	2.41 ± 1.28	1.27 ± 1.30
Cortisol, mg/dL	17.22 ± 16.97	9.07 ± 6.48
Testosterone, nmol/L	4.75 ± 5.45	5.97 ± 4.65
FSH, mUl/mL	5.90 ± 2.38	1.40 ± 0.83
LH, mUl/mL	3.84 ± 1.87	0.99 ± 0.96

As expected, there was a significant reduction in the volume of the tumor after treatment with cabergoline. The Pearson correlation test revealed that pre-treatment tumor volume correlated with baseline sexual dysfunction (high-degree correlation coefficient: 0.829, p=0.003 with CSFQ score) and impulsivity at six months (high-degree correlation coefficient: 0.794, p=0.034 with BIS score). The sum of affected areas on baseline MRI also correlated with baseline sexual dysfunction. No significant correlation was found between tumor volume after treatment and any other variable. Finally, a marked decrease in prolactin levels and a downward trend in TSH, cortisol, FSH, and LH were observed, along with an upward trend in testosterone levels at the end of the follow-up. Despite the recovery of the hormonal axes, the patients did not experience significant changes in the CSFQ scale, as described in Table 4.

**Table 4:** Pre-treatment and post-treatment (six months) scale scores *Statistically significant at p<0.05 SD: standard deviation; BIS: Barrat Impulsiveness Scale; AHS: Anxiety Hamilton Scale; HDS: Hamilton Depression Scale; CSFQ: Changes in Sexual Functioning Questionnaire

Measure/scale	Pre-treatment, mean score ± SD	Post-treatment, mean score ± SD	P-value*
BIS	49.90 ± 15.61 (n=10)	48.25 ± 15.69 (n=8)	0.031
HDS	13.20 ± 7.40 (n=10)	7.37 ± 9.05 (n=8)	0.083
AHS	18.10 ± 9.20 (n=10)	16 ± 12.4 (n=8)	0.199
CSFQ	39 ± 13.9 (n=10)	43.1 ± 9.1 (n=8)	0.201
Tumoral volume	36.20 ± 29.78 (n=10)	7.32 ± 12.7 (n=9)	0.002

## Discussion

Our study reported an incidence rate of neuropsychiatric adverse effects of 20%, including one case of mania with psychotic symptoms and one case of hypersexuality, both observed in male subjects. These findings are consistent with the incidence of impulse control disorders reported by other authors, which ranged from 8% to 20% [6-12]. The patient with the most severe neuropsychiatric symptoms had the largest tumor volume and the most invasive tumor behavior among the sample, which also affected his prognosis. He developed medical complications such as CSF fistula and meningoencephalitis and ultimately died. We propose that the severity of the underlying disease is positively correlated with the probability of experiencing medical and neuropsychiatric complications.

Of note, 70% of the patients were diagnosed with depression during the first psychiatric evaluation. Before starting treatment, these patients were classified as having mild to moderate depression severity. Hyperprolactinemia could explain this symptomatology as it downregulates dopamine synthesis and the hypothalamus-pituitary-adrenal axis, resulting in various effects on testosterone levels. Evidence suggests that male patients with hypogonadism exhibit more depressive symptoms than males with normal levels of sexual hormones [13-29], which may contribute to a multifactorial model as a cause of depression in our patients.

A second mechanism may be associated with the use of dopaminergic agonists, which affect the mesocorticolimbic pathway and the reward system. Evidence suggests that the chronic use of dopaminergic agonists can alter reward-linked learning, leading to failures in the prediction processing system and the activation of reward systems. This, in turn, increases the subjective experience of possible rewards, making them more stimulating than in subjects without dopaminergic agonist intake [30]. Additionally, no significant improvement was observed in the scores of the sexual function scales throughout the follow-up period. It must be kept in mind that even though cabergoline was the main treatment given to these patients, other interventions such as antidepressants and cognitive behavioral therapy were offered when appropriate. 

During follow-up, our patients reported clinical improvement in neuropsychiatric symptoms, including anxiety; however, no significant improvement in HAM-A scores was observed. This may be due to the sample size. Neurological symptoms attributed to the mass effect also improved. This study provides relevant information regarding the epidemiology of neuropsychiatric manifestations in patients with invasive prolactinomas, obtained prospectively. It also looks into hypotheses about the possible mechanisms by which cabergoline treatment may influence these symptoms.

The following limitations of the study must be taken into account when interpreting the results. Even though we calculated a sample size of 15, only 10 patients were ultimately recruited. Furthermore, we lost one patient during follow-up; he died of complications related to the tumor. However, it only represents 10% of our sample. Additionally, the follow-up period may have fallen short in terms of treatment response regarding neuropsychiatric symptoms, since it is known that many of the benefits of treatment become evident only after one year of follow-up. All patients included were Mexican, which could have led to an ethnic bias as well. This study was conducted at the National Institute of Neurology and Neurosurgery in Mexico City, which is a public hospital depending solely on the government budget, limiting the resources available for treatment and follow-up.

## Conclusions

Invasive prolactinomas are associated with significant neuropsychiatric symptoms that may cause a major burden on the quality of life of the patients. Our findings suggest that tumor volume correlates with baseline sexual dysfunction and anxiety upon follow-up. These symptoms could be attributed to the underlying disease mechanisms, functional impairment, the high incidence of neurological comorbidity, or the treatment with cabergoline. It is of utmost importance to screen these patients for these comorbidities with standardized clinical scales to ensure that adequate multidisciplinary treatment is provided. The role of cabergoline in these symptoms remains unexplored; this study shows that two out of 10 patients developed neuropsychiatric symptoms after treatment was started, and hence further studies to clarify whether the benefits on tumor control outweigh the potential psychiatric adverse effects due to disruption of mesolimbic and mesocortical dopaminergic pathways are needed.
